# Supplementary material for: Molecular dynamics simulations of the evaporation of hydrated ions from aqueous solution
Source: Commun Chem. 2022 Apr 19;5:55. doi: 10.1038/s42004-022-00669-5 (PMC9814746; doi:10.1038/s42004-022-00669-5)
Supplement: Supplementary file 2 — Supplemental Information [file 42004_2022_669_MOESM2_ESM.pdf]

Molecular Dynamics Simulations of the Evaporation of Hydrated Ions from Aqueous Solution: Supporting Information

Philip Loche,[1,2] Douwe J. Bonthuis,[3] and Roland R. Netz[2,*]

[1]*Laboratory of Computational Science and Modeling, IMX, École Polytechnique Fédérale de Lausanne, 1015 Lausanne, Switzerland*
[2]*Fachbereich Physik, Freie Universität Berlin, 14195 Berlin, Germany*
[3]*Institute of Theoretical and Computational Physics, Graz University of Technology, 8010 Graz, Austria*

CONTENTS

Supplementary Section 1. Details of simulation and TI methods 1

Supplementary Section 2. Constant velocity simulations 2

Supplementary Section 3. Electrostatic energies in 2D periodic interface system 2

Supplementary Section 4. Comparison to results from umbrella sampling methods 6

Supplementary Section 5. Rate theory in the presence of a reservoir 7

Supplementary Section 6. Reaction rates are dominated by the transition-state diffusivity 9

Supplementary References 10

Supplementary Section 1. Details of simulation and TI methods

During the performed molecular dynamics simulations the water bond lengths of the SPC/E water model are constrained using the SETTLE algorithm[1] and the integration time step is set to 2 fs. 2-dimensional long-range electrostatics are handled using the smooth particle mesh Ewald (SPME) technique with a slab correction according to Ref.[2]. Lennard-Jones interactions are cut off at a distance 0.9 nm without dispersion correction.

The chloride Lennard-Jones parameters ($\sigma = 0.452\,\mathrm{nm}$, $\epsilon = 0.4186\,\mathrm{kJ \cdot mol^{-1}}$) are taken from Ref. 3. Supplementary Figure 1 shows the chloride-oxygen radial distribution function, where B_O is the position of the first peak used for

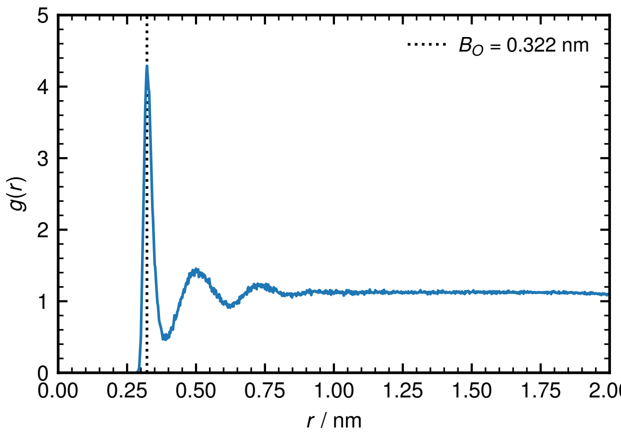

Supplementary Figure 1. Chloride-oxygen radial distribution function.

* rnetz@physik.fu-berlin.de

estimating the binding energy given in Eq. (4) in the main text. For the positively charged counter ion, we choose Lennard-Jones parameters for a lithium ion ($\sigma = 0.2\,\mathrm{nm}$, $\epsilon = 0.4186\,\mathrm{kJ\cdot mol^{-1}}$), so that the solvation free energy has a larger magnitude compared to the negative chloride ion. The negative chloride has a solvation free energy of $-135.89\,\mathrm{kJ\cdot mol^{-1}}$ and the positive counter ion of $-162.2\,\mathrm{kJ\cdot mol^{-1}}$. Production runs of all systems are performed in the NVT-ensemble at $T = 300\,\mathrm{K}$ using a velocity rescale thermostat including a stochastic factor[4] with a time constant of 0.5ps. The partial pressure of the water vapor is $P_\mathrm{vap} = 30.12\,\mathrm{mbar}$ which is obtained from the average particle number $N_\mathrm{avg} = 1.13$ in the vapor phase and $V = 1604\,\mathrm{nm}^3$, the volume of the vapor phase, by using the ideal gas law

$$P_\mathrm{vap} = \frac{N_\mathrm{avg} k_\mathrm{B} T}{V}\,. \tag{1}$$

This value is in agreement with values reported previously[5] and corresponds to roughly 70% of the experimental value[6]. The thermodynamic integration (TI) calculations of the ion free energy are done in two steps: First all Lennard-Jones interactions between the chloride, the counter ion, and all other atoms are gradually turned on; second, the charges of the chloride and the counterion are gradually increased from $q = 0$ to $\pm 1e$. The integration is performed along an alchemical reaction coordinate λ, where $\lambda = 0$ corresponds to the initial (A) and $\lambda = 1$ to the final state (B). For the integration the Hamiltonian is interpolated linearly $H = (1-\lambda)H_A + \lambda H_B$. Free energy differences are calculated by integrating $\langle \partial H/\partial\lambda\rangle$ from $\lambda = 0$ to $\lambda = 1$ using the alchemical-analysis toolkit[7]. The Lennard-Jones transformation is divided into 10 steps and the charging transformation into 22. For the integration of the Lennard-Jones potential, we use a soft-core potential to prevent a singularity at $\lambda = 0$ with a soft-core radius $\alpha = 0.5\,\mathrm{nm}$ and a soft-core power $p = 1.27$[8]. The simulation time for each λ state is at least 5 ns and up to 100 ns, where we use the first 500 ps of the short simulations and the first 50 ns of the long simulations for equilibration. Errors of the TI results are obtained by calculating the mean and the standard error of the individual integration steps using uncorrelated samples[7,9]. The error of the integrated free energy is then calculated using error propagation of the trapezoidal integration rule. With this, we estimate an error of the TI results for the chloride free energy of $\approx 1\,\mathrm{k_BT}$, which is smaller than the symbol sizes used in the plots. However, a larger error of the TI data of roughly $5\,\mathrm{k_BT}$ can be estimated from the scattering among the TI results or from the deviation of the TI data from the cubic model fit. That the latter error is larger than the error estimate for the individual TI results presumably reflects the slow water finger and water hydration dynamics, which is not accounted for in the TI results for a fixed separation from the interface. For calculating the Gibbs dividing surface we use

$$z^{GDS} = z_I + \int_{z_I}^{z_{II}} \frac{\rho_m(z_{II}) - \rho_m(z)}{\rho_m(z_{II}) - \rho_m(z_I)}\mathrm{d}z, \tag{2}$$

where $\rho_m(z)$ is the mass density profile and z_I and z_{II} are two positions in the two bulk phases. Due to the formation of the water finger, we calculate the dividing surfaces for every position of the chloride ion individually.

Supplementary Section 2. Constant velocity simulations

Similar to Fig. 1 in the main text, Supplementary Figure 2 shows the ion position z and the number of water molecules $N_{\mathrm{H_2O}}$ for simulations where the ion is confined in a potential that is moved at different constant velocities.

Supplementary Section 3. Electrostatic energies in 2D periodic interface system

We now estimate the total electrostatic energy U_tot for two ions (A, B) in a 2D periodic interface system. We divide the system into two regions $i = 1, 2$ each with a different dielectric constant ε_i and divided by a sharp and flat interface. The total energy is given by

$$U_\mathrm{tot} = \sum_{n_x,n_y=-N_\mathrm{images}}^{N_\mathrm{images}} \int \rho(\boldsymbol{r})\mathcal{G}(\boldsymbol{r},\boldsymbol{r}' + \boldsymbol{L}\cdot\boldsymbol{n})\rho(\boldsymbol{r}' + \boldsymbol{L}\cdot\boldsymbol{n})\mathrm{d}\boldsymbol{r}\mathrm{d}\boldsymbol{r}'\,, \tag{3}$$

where $\boldsymbol{L}$ is a diagonal matrix with elements L_x, L_y, L_z, the dimensions of the rectangular unit cell. $\boldsymbol{n} = (n_x, n_y, 0)$ is a vector with integer components n_x, n_y and $\rho(\boldsymbol{r})$ is the charge density. If not stated otherwise we treat the charge density as a sum of two delta peaks

$$\rho(\boldsymbol{r}) = q_A\delta(\boldsymbol{r} - \boldsymbol{r}_A) + q_B\delta(\boldsymbol{r} - \boldsymbol{r}_B)\,, \tag{4}$$

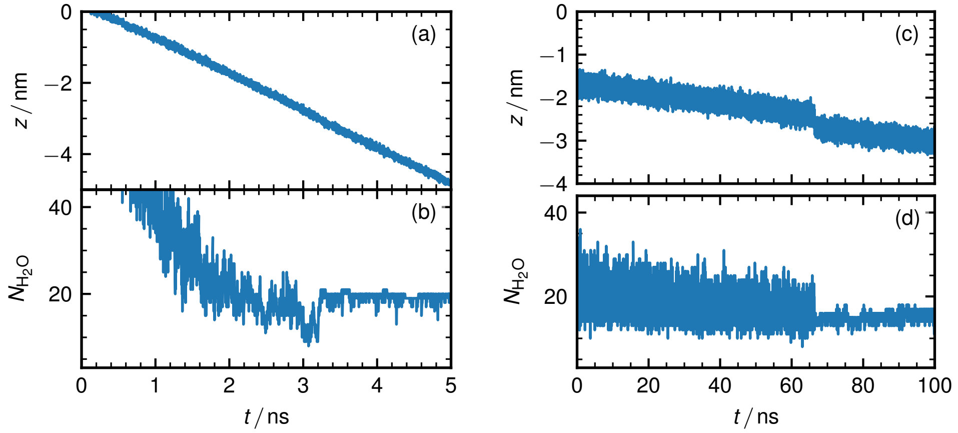

Supplementary Figure 2. Ion position z and number of water molecules $N_{\mathrm{H_2O}}$ in a spherical test shell of 1 nm radius around the ion. The ion is held in a harmonic potential with a force constant of $k = 1000\mathrm{kJ} \cdot \mathrm{mol}^{-1} \cdot \mathrm{nm}^{-2}$ moving with a constant velocity. a–b show results for a velocity of $v = 1\,\mathrm{m} \cdot \mathrm{s}^{-1}$ and c–d show results for $v = 10^{-2}\,\mathrm{m} \cdot \mathrm{s}^{-1}$.

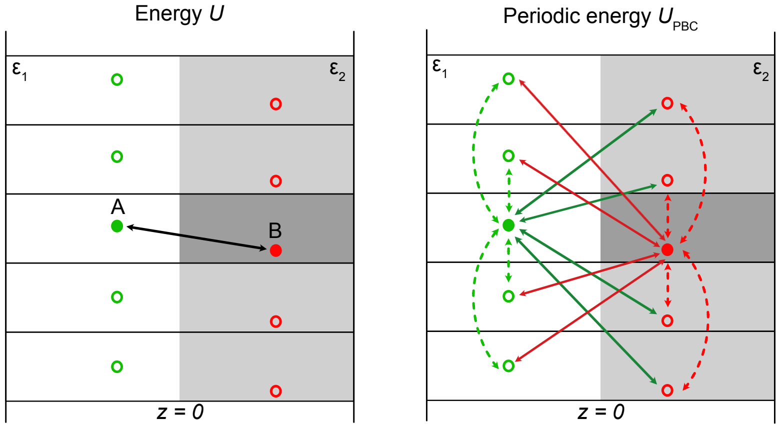

Supplementary Figure 3. Contributions to the electrostatic energy of the chloride ion (A) and the counter ion (B) in a 2D periodic interface system. U is the interaction inside the primary unit cell. U_{PBC} is the interaction energy of the ions in the primary cell with their images. The interface is positioned at the Gibbs dividing surface at $z = 0$.

where q_A and q_B are the charges of the two ions and $\boldsymbol{r}_A$ and $\boldsymbol{r}_B$ are their positions. We subtract the diverging diagonal term of the interaction implicitly. For the first $\rho(\boldsymbol{r})$ in Supplementary Equation (3) we do not sum over the periodic images since we are interested in the energy due to interactions of the charges in the simulation box with their periodic images, not due to interactions between the periodic images. This becomes more clear when splitting off the term where $n_x = n_y = 0$

$$U_{\mathrm{tot}} = \frac{1}{2} \int \rho(\boldsymbol{r}) \mathcal{G}(\boldsymbol{r}, \boldsymbol{r}') \rho(\boldsymbol{r}') \mathrm{d}\boldsymbol{r} \mathrm{d}\boldsymbol{r}' + \sum_{\substack{n_x, n_y = -N_{\mathrm{images}} \\ n_x, n_y \neq 0}}^{N_{\mathrm{images}}} \int \rho(\boldsymbol{r}) \mathcal{G}(\boldsymbol{r}, \boldsymbol{r}' + \boldsymbol{L} \cdot \boldsymbol{n}) \rho(\boldsymbol{r}' + \boldsymbol{L} \cdot \boldsymbol{n}) \mathrm{d}\boldsymbol{r} \mathrm{d}\boldsymbol{r}' \tag{5}$$

$$:= U + U_{\mathrm{PBC}} \,. \tag{6}$$

The term where $n_x = n_y = 0$ is called U and denotes the interaction in the primary unit cell, where the factor of $1/2$ avoids overcounting. All terms where $n_x \neq 0$ and $n_y \neq 0$ are interactions of the simulation box with the periodic images and are denoted by U_{PBC}. In Supplementary Figure 3 we show a representation of the setup. The interaction in the primary unit cell consists of three contributions

$$U = U_{\mathrm{self}}^{(A)} + U_{\mathrm{self}}^{(B)} + U_{\mathrm{A,B}} \,. \tag{7}$$

The first two terms are the self energies of the ions A and B, respectively. The last term is the direct interaction between the two ions. $U_{\mathrm{A,B}}$ was derived previously[10,11] and is of the order of one $\mathrm{k_B T}$ for our system and thus is irrelevant. $U_{\mathrm{self}}^{(B)}$ has the same structure as $U_{\mathrm{self}}^{(A)}$ given below in Supplementary Equation (8). However, ion B has a constant position at all times and therefore its energy is constant and irrelevant as well.

For $U_{\mathrm{self}}^{(A)}$ and for $U_{\mathrm{self}}^{(B)}$ we describe the ions by charge distributions corresponding to uniformly charged spherical shells with a surface charge density $\sigma = q/(4\pi R^2)$, where R is the radius of the sphere. The dielectric constant inside the spheres is the same as of the environment. For a single ion, i.e. ion A, at a sharp dielectric interface (neglecting

the finite thickness of the water slab), $U_{\rm self}$ was previously derived[10] and reads

$$U_{\rm self}^{(A)}(x) := \frac{q_A^2}{8\pi\varepsilon_0\varepsilon_2 R}\, u_{\rm self}(x) \tag{8}$$

where $x = z/R$ and

$$u_{\rm self}(x \geq 1) = 1 + \frac{\lambda}{2x} \tag{9}$$

$$u_{\rm self}(|x| < 1) = \left(\frac{1}{1-\lambda}\right)\left\{1 - \frac{\lambda x}{2}(1-\lambda) - \lambda^2\left[I_A(x) + I_B(x)\right]\right\} \tag{10}$$

$$u_{\rm self}(x \leq -1) = \left(\frac{1+\lambda}{1-\lambda}\right)\left(1 + \frac{\lambda}{2x}\right)\,. \tag{11}$$

Here $\lambda = (\epsilon_2 - \epsilon_1)/(\epsilon_1 + \epsilon_2)$ is the dielectric contrast and

$$I_A(x) = \frac{1}{\pi\sqrt{2}}\int_{-x}^{1} \mathrm{d}(\cos\theta)\int_{-1}^{x}\mathrm{d}(\cos\theta')\frac{K\left[\frac{2\sin\theta\sin\theta'}{1-\cos(\theta+\theta')+2x(\cos\theta-\cos\theta'+x)}\right]}{\sqrt{1-\cos(\theta+\theta')+2x(\cos\theta-\cos\theta'+x)}} \tag{12}$$

$$I_B(x) = \frac{1}{\pi\sqrt{2}}\int_{-x}^{1} \mathrm{d}(\cos\theta)\int_{-1}^{x}\mathrm{d}(\cos\theta')\frac{K\left[\frac{2\sin\theta\sin\theta'}{1+\cos(\theta-\theta')}\right]}{\sqrt{1+\cos(\theta-\theta')}} \tag{13}$$

$$\tag{14}$$

are integrals in terms of the complete elliptic integral of the first kind,

$$K[m] := \int_0^{\pi/2}\frac{\mathrm{d}\alpha}{\sqrt{1-m^2\sin^2\alpha}}\,. \tag{15}$$

We now derive the second part of Supplementary Equation (5), namely the interactions of the two ions with their periodic replica. To obtain this contribution, we use previously derived Green's functions and treat the ions as point charges. Therefore, the periodic interaction are given by

$$U_{\rm PBC} = \frac{qq'}{2}\sum_{\substack{n_x,n_y=-N_{\rm images}\\ n_x,n_y\neq 0}}^{N_{\rm images}} \mathcal{G}(\boldsymbol{r}, \boldsymbol{r}' + \boldsymbol{L}\cdot\boldsymbol{n})\,. \tag{16}$$

In the following, $\mathcal{G}_i^j(\boldsymbol{r},\boldsymbol{r}')$ defines Green's functions where i denotes the region of the observer at position $\mathbf{r}$ and j the region of the charge at position $\mathbf{r}'$. The Green's functions are derived in Ref. 10 and read, if the charge is in region 1 at $z < 0$ and the observer is also in region 1,

$$\mathcal{G}_1^1(\boldsymbol{r},\boldsymbol{r}') = \frac{1}{4\pi\varepsilon_0}\frac{1}{\varepsilon_1}\left(\frac{1}{|\boldsymbol{r}-\boldsymbol{r}'|} - \frac{\lambda}{|\boldsymbol{r}-(\boldsymbol{r}'-2\boldsymbol{r}'\hat{z})|}\right) \tag{17}$$

where $\hat{z}$ is the unit vector in the z direction. $|\boldsymbol{r}-\boldsymbol{r}'|$ is the distance from the observer relative to the ion and $|\boldsymbol{r}-(\boldsymbol{r}'-2\boldsymbol{r}'\hat{z})|$ the distance from the observer to the image charge created by the interface at $z = 0$. If the charge is in region 1 and the observer is in region 2 one finds

$$\mathcal{G}_1^2(\boldsymbol{r},\boldsymbol{r}') = \frac{1}{4\pi\varepsilon_0\varepsilon_1}\frac{1+\lambda}{|\boldsymbol{r}-\boldsymbol{r}'|}\,. \tag{18}$$

If the charge and the observer are in region 2 the Green's function reads

$$\mathcal{G}_2^2(\boldsymbol{r},\boldsymbol{r}') = \frac{1}{4\pi\varepsilon_0\varepsilon_2}\left(\frac{1}{|\boldsymbol{r}-\boldsymbol{r}'|} + \frac{\lambda}{|\boldsymbol{r}-(\boldsymbol{r}'-2\boldsymbol{r}'\hat{z})|}\right) \tag{19}$$

and if the charge is in region 2 and the observer in region 1 we have

$$\mathcal{G}_2^1(\boldsymbol{r},\boldsymbol{r}') = \frac{1}{4\pi\varepsilon_0\varepsilon_2}\frac{1-\lambda}{|\boldsymbol{r}-\boldsymbol{r}'|}\,. \tag{20}$$

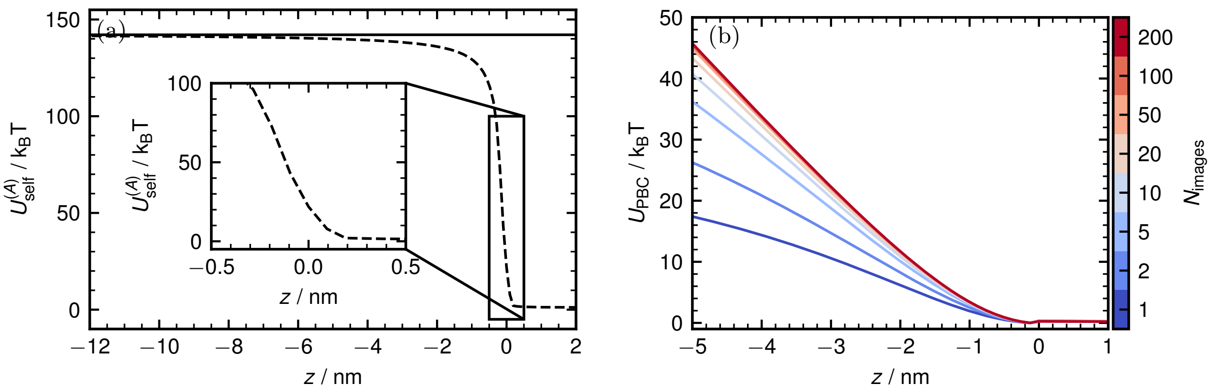

Supplementary Figure 4. (a): $U_{\text{self}}^{(A)}$ as given by Supplementary Equation (8) for an ion radius $R = 0.19\,\text{nm}$. (b): U_{PBC} as given by Supplementary Equation (16) as a function of the number of periodic images N_{images}.

If both ions are in region 2, the periodic interactions are given by

$$\begin{aligned} U_{\text{PBC}}(x>0) = {} & \frac{q_A^2}{2} \sum_{\substack{n_x,n_y=-N_{\text{images}} \\ n_x,n_y\neq 0}}^{N_{\text{images}}} \mathcal{G}_2^2(\boldsymbol{r}_A, \boldsymbol{r}_A + \boldsymbol{L}\cdot\boldsymbol{n}) + \frac{q_B^2}{2} \sum_{\substack{n_x,n_y=-N_{\text{images}} \\ n_x,n_y\neq 0}}^{N_{\text{images}}} \mathcal{G}_2^2(\boldsymbol{r}_B, \boldsymbol{r}_B + \boldsymbol{L}\cdot\boldsymbol{n}) \\ & + \frac{q_A q_B}{2} \sum_{\substack{n_x,n_y=-N_{\text{images}} \\ n_x,n_y\neq 0}}^{N_{\text{images}}} \mathcal{G}_2^2(\boldsymbol{r}_A, \boldsymbol{r}_B + \boldsymbol{L}\cdot\boldsymbol{n}) + \frac{q_B q_A}{2} \sum_{\substack{n_x,n_y=-N_{\text{images}} \\ n_x,n_y\neq 0}}^{N_{\text{images}}} \mathcal{G}_2^2(\boldsymbol{r}_B, \boldsymbol{r}_A + \boldsymbol{L}\cdot\boldsymbol{n}) \,, \end{aligned} \tag{21}$$

where $\boldsymbol{r}_A$ and $\boldsymbol{r}_B$ are the positions of the two ions. If ion A is in region 1 and ion B in region 2, the periodic interactions are given by

$$\begin{aligned} U_{\text{PBC}}(x<0) = {} & \frac{q_A^2}{2} \sum_{\substack{n_x,n_y=-N_{\text{images}} \\ n_x,n_y\neq 0}}^{N_{\text{images}}} \mathcal{G}_1^1(\boldsymbol{r}_A, \boldsymbol{r}_A + \boldsymbol{L}\cdot\boldsymbol{n}) + \frac{q_B^2}{2} \sum_{\substack{n_x,n_y=-N_{\text{images}} \\ n_x,n_y\neq 0}}^{N_{\text{images}}} \mathcal{G}_2^2(\boldsymbol{r}_B, \boldsymbol{r}_B + \boldsymbol{L}\cdot\boldsymbol{n}) \\ & + \frac{q_A q_B}{2} \sum_{\substack{n_x,n_y=-N_{\text{images}} \\ n_x,n_y\neq 0}}^{N_{\text{images}}} \mathcal{G}_2^1(\boldsymbol{r}_A, \boldsymbol{r}_B + \boldsymbol{L}\cdot\boldsymbol{n}) + \frac{q_B q_A}{2} \sum_{\substack{n_x,n_y=-N_{\text{images}} \\ n_x,n_y\neq 0}}^{N_{\text{images}}} \mathcal{G}_1^2(\boldsymbol{r}_B, \boldsymbol{r}_A + \boldsymbol{L}\cdot\boldsymbol{n}) \,. \end{aligned} \tag{22}$$

Supplementary Equations (21) and (22) are evaluated numerically. Supplementary Figure 4 shows $U_{\text{self}}^{(A)}$ and U_{PBC}, where $U_{\text{self}}^{(A)}$ is also shown in Fig. 3b in the main text and in Supplementary Figure 6b. The plot demonstrates that the image charge potential slowly saturates, since $U_{\text{self}}^{(A)} \propto z^{-1}$ as given in Supplementary Equation (8). Supplementary Figure 4b shows U_{PBC} as a function of the number of images N_{images}. Here ion B is fixed at $z = 5$ and ion A is moved along the z axis across the interface. The unit cell dimensions are $L_x = 5.1\,\text{nm}$ and $L_y = 5.4\,\text{nm}$. As shown, the energy converges for $N_{\text{images}} > 50$.

So far in our derivation, we used a model containing a single interface only, whereas in the simulations we have a slab system with a thickness of 10 nm containing two interfaces. Two account for the presence of two interfaces, we insert the Green's functions for a slab system from our previous work[11] into Supplementary Equation (22). In Supplementary Figure 5 we compare U_{PBC} for a slab system for a few different slab thicknesses L for $N_{\text{images}} = 50$. We find that for a thickness of $L = 10\,\text{nm}$, the electrostatic energy for a slab system agrees with the result for a single interface shown in Supplementary Figure 4b and shown as a black dashed line in Supplementary Figure 5. Therefore, our usage of a model containing only single-interface Green's functions to correct the simulation results in the main text is justified.

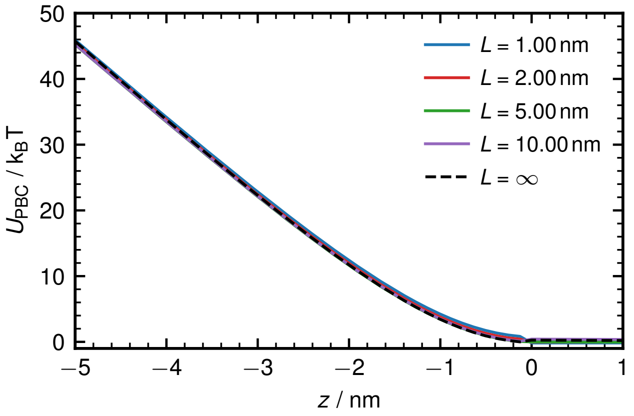

Supplementary Figure 5. U_{PBC} as a function of z for a few different water-slab thicknesses L where $N_{\text{images}} = 50$. The black dashed line corresponds to the solution for a single interface obtained in the limit $L \to \infty$ as shown in Supplementary Figure 4b. Green's functions for the slab system are taken from our previous work[11].

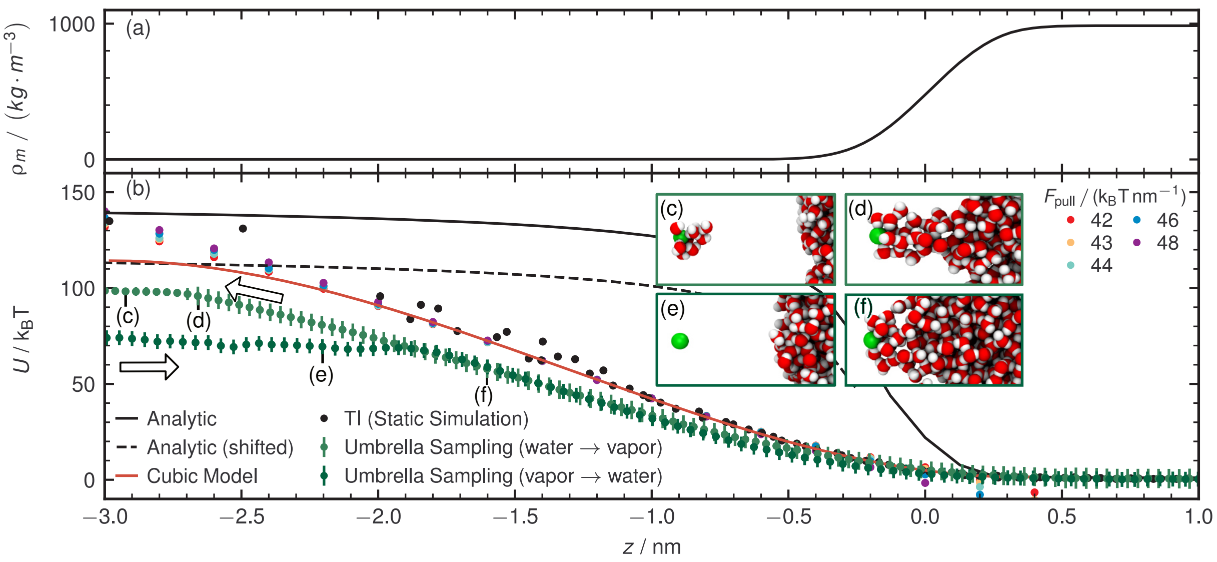

Supplementary Figure 6. (a): Water density profile $\rho_m(z)$. (b): Free energy profile $U(z)$ from thermodynamic integration (TI, black dots, same data as shown in the main text) and from umbrella sampling (dark and light green data points). For the umbrella sampling method the ion is confined in umbrella potentials with a force constant $1000\,\text{kJ} \cdot \text{mol}^{-1} \cdot \text{nm}^{-2}$ and the starting configurations come from pull simulations where the ions are moved towards the water phase (dark green) or towards the vapor phase (light green) with a velocity of $1\,\text{m} \cdot \text{s}^{-1}$. (c)–(f): Snapshots at different ion positions as indicated in (b).

Supplementary Section 4. Comparison to results from umbrella sampling methods

In Supplementary Figure 6b we compare the free energy $U(z)$ obtained from TI and umbrella sampling. The black dots show the results from the TI from the main text in Figure 3b. The green dots show the results from umbrella sampling with equally spaced umbrella potentials at a distance of 0.2 nm, going from the vapor into the liquid and going from the liquid into the vapor. We obtain the initial configurations for each simulation series from a pull simulation starting in the water or in the vapor phase, respectively, with a pulling velocity of the harmonic potential of $1\,\text{m} \cdot \text{s}^{-1}$ and a force constant of $1000\,\text{kJ} \cdot \text{mol}^{-1} \cdot \text{nm}^{-2}$. The free energy from the umbrella sampling simulations is obtained by using the Weighted Histogram Analysis Method (WHAM)[12]. The spacing of the green data points (0.05 nm) shown in Supplementary Figure 6b results by post-processing from the WHAM method. From all simulated free energy profiles, we subtract the effect of the periodic images $U_{\text{PBC}}(z)$ as discussed in section Supplementary Section 3. Errors of the umbrella sampling data points are obtained using a bootstrap analysis[12] using 100 bootstraps and are of the order of $5\,\text{k}_\text{B}\text{T}$. We suspect that the actual error of the umbrella sampling simulations is much larger and in fact accounts for the deviations from the TI results, since the umbrella sampling results that start in the vapor phase do not converge to the free energy of a naked, unhydrated ion for large separations from the interface, as given by the analytical prediction for the image charge free energy (solid black line). This to a certain degree is due to the

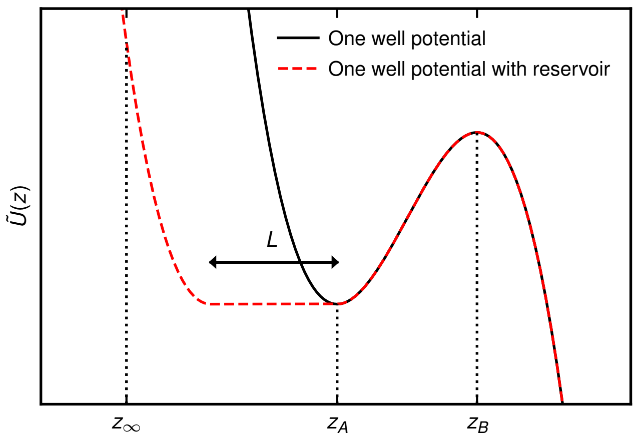

Supplementary Figure 7. Schematic representation of a single-well potential without and with a reservoir of length L.

fact that we invest less computational effort in the free energy calculations using the umbrella method compared to TI. Simulations are run for 5 ns for each umbrella potential position, whereas for the TI data we use up to 20 times longer trajectories for each integration step, in particular for large distances from the interface for $z < -1.5\,\mathrm{nm}$. We suspect that the main difficulty of the umbrella results to reproduce the free energy for unhydrated ions far away from the interface is related to the WHAM procedure, which tends to flatten the global free energy landscape. Therefore, we do not use the umbrella sampling results for our analysis in the main text. The insets (Supplementary Figure 6c–f) show snapshots at different ion positions. The free energy $U(z)$ from umbrella sampling also exhibits pronounced hysteresis for large separations from the interface, which originates from the slow relaxation of the hydration structure around the ion.

Supplementary Section 5. Rate theory in the presence of a reservoir

In the following, we derive the barrier-crossing rate for a particle in a potential well in the presence of a bulk reservoir. See Supplementary Figure 7 for a schematic representation. The mean first passage time τ_{MFP} for starting at position z_0 near the potential minimum z_A and reaching position z_B at the potential maximum for the first time is given by[13]

$$\tau_{\mathrm{MFP}} = \frac{1}{D_{\mathrm{tr}}}\int_{z_0}^{z_B} \mathrm{d}z \mathrm{e}^{\tilde{U}(z)} \int_{z_\infty}^{z} \mathrm{d}\tilde{z}\mathrm{e}^{-\tilde{U}(\tilde{z})} = \frac{1}{D_{\mathrm{tr}}}\int_{z_0}^{z_B} \mathrm{d}z \mathrm{e}^{\tilde{U}(z)} I(z)\,. \tag{23}$$

Here D_{tr} is the diffusion constant in the transition state and $\tilde{U}(z) = U(z)/k_{\mathrm{B}}T$ is the rescaled potential. At z_∞ the potential is assumed to reach infinity $\tilde{U}(z) = \infty$. For solving the integral given in Supplementary Equation (23) we expand the function $I(z)$ around the maximum of the potential at z_B

$$I(z) = \int_{z_\infty}^{z} \mathrm{d}z \mathrm{e}^{-\tilde{U}(z)} = I(z_B) + (z - z_B)I'(z_B) + \frac{1}{2}(z - z_B)^2 I''(z_B)\,. \tag{24}$$

We approximate the first term in Supplementary Equation (24) by

$$I(z_B) = \int_{z_\infty}^{z_B} \mathrm{d}z \mathrm{e}^{-\tilde{U}(z)} \approx \int_{-\infty}^{\infty} \mathrm{d}z \mathrm{e}^{-\tilde{U}(z)}\,, \tag{25}$$

noting that the main contribution comes from the region around z_A. The derivatives of $I(z_B)$ are

$$I'(z_B) = \mathrm{e}^{-\tilde{U}(z_B)} \qquad\qquad I''(z_B) = -\tilde{U}'(z_B)\mathrm{e}^{-\tilde{U}(z_B)}\,. \tag{26}$$

Putting Supplementary Equations (25) and (26) into the expression for the inner integral as given in Supplementary Equation (24) we obtain

$$I(z) = I(z_B) + (z - z_B)\mathrm{e}^{-\tilde{U}_B} - \frac{1}{2}(z - z_B)^2 \tilde{U}'_B \mathrm{e}^{-\tilde{U}_B}\,, \tag{27}$$

where $\tilde{U}_B$ and $\tilde{U}'_B$ are the potential and its first derivative at z_B. To estimate $I(z_B)$ we expand $\tilde{U}(z)$ around the minimum at z_A up to second order

$$\tilde{U}(z) \approx \tilde{U}_A + \frac{1}{2}(z - z_A)^2 \tilde{U}''_A \,. \tag{28}$$

Note that in the upper equation (28) there is no linear term, since $\tilde{U}'(z_A) = 0$. Inserting (28) into Supplementary Equation (25) we find

$$\begin{aligned} I(z_B) &= \mathrm{e}^{-\tilde{U}_A} \int_{-\infty}^{\infty} \mathrm{d}z \mathrm{e}^{\frac{1}{2}(z-z_A)^2 U''_A} \\ &= \mathrm{e}^{-\tilde{U}_A} \sqrt{\frac{2\pi}{U''_A}} \,. \end{aligned} \tag{29}$$

With the latter expansion Supplementary Equation (27) further simplifies to

$$I(z) = \mathrm{e}^{-\tilde{U}_A} \left[\sqrt{\frac{2\pi}{U''_A}} - \mathrm{e}^{-\left(\tilde{U}_B - \tilde{U}_A\right)} (z - z_B) \right] , \tag{30}$$

where the third term in Supplementary Equation (27) vanishes since $\tilde{U}'_B = 0$. Note that the second term in the brackets vanishes for large barrier height. We now account for the reservoir of length L and write the inner integral as

$$I(z) \approx I(z_B) = \mathrm{e}^{-\tilde{U}_A} \left[\sqrt{\frac{2\pi}{U''_A}} + L \right] . \tag{31}$$

Now the inner integral does not depend on z so the nested integral in Supplementary Equation (23) factorizes and we can write

$$\begin{aligned} I_{\mathrm{out}}(z) &= \int_{z_0}^{z_B} \mathrm{d}z \mathrm{e}^{\tilde{U}(z)} \approx \frac{1}{2} \int_{-\infty}^{\infty} \mathrm{d}z \mathrm{e}^{\tilde{U}_B - \frac{1}{2}(z-z_B)^2 |U''_B|} \\ &= \frac{1}{2} \mathrm{e}^{\tilde{U}_B} \sqrt{\frac{2\pi}{|U''_B|}} . \end{aligned} \tag{32}$$

The factor 1/2 stems from the extensions of the integral boundaries while using the symmetry of the integral. We now insert Supplementary Equations (31) and (32) into τ_{MFPT} as given in Supplementary Equation (23) and find

$$\tau_{\mathrm{MFP}} = \frac{1}{D_{\mathrm{tr}}} \sqrt{\frac{\pi}{2\tilde{U}''_B}} \left(\sqrt{\frac{2\pi}{\tilde{U}''_A}} + L \right) \mathrm{e}^{\tilde{U}_B - \tilde{U}_A} \,. \tag{33}$$

The escape rate over the maximum is defined as the inverse of $2\tau_{\mathrm{MFP}}$

$$k := \frac{1}{2\tau_{\mathrm{MFPT}}} = \frac{k_0}{\sqrt{\frac{2\pi}{\tilde{U}''_A}} + L} \mathrm{e}^{-\Delta \tilde{U}} \,, \tag{34}$$

with $k_0 = D_{\mathrm{tr}} \sqrt{\tilde{U}''_B / (2\pi)}$ being the rate coefficient with units of a velocity and $\Delta\tilde{U} = \tilde{U}_B - \tilde{U}_A$ is the rescaled barrier height. The limiting case for $L \to \infty$ is

$$\lim_{L\to\infty} k = \frac{D_{\mathrm{tr}}}{L} \sqrt{\frac{\tilde{U}''_B}{2\pi}} \mathrm{e}^{-\Delta\tilde{U}} = \frac{k_0}{L} \mathrm{e}^{-\Delta\tilde{U}} \tag{35}$$

and for $L \to 0$ we recover the standard Kramers rate given by

$$\lim_{L\to 0} k = \frac{D_{\mathrm{tr}}}{2\pi} \sqrt{\tilde{U}''_B \tilde{U}''_A} \mathrm{e}^{-\Delta\tilde{U}} \,. \tag{36}$$

Supplementary Table I. Optimal fit parameters of the cubic polynomial fit: $U(z) = az^3 + bz^2 + cz + d$ shown in Fig. 3 in the main text. Values for the barrier height ΔU are calculated according to Supplementary Equation (39), the curvature at the extrema $U''_{\mathrm{A,B}}$ according to Supplementary Equation (40) is given as well.

$a\,/\,\mathrm{k_B T}\cdot\mathrm{nm}^{-3}$	$b\,/\,\mathrm{k_B T}\cdot\mathrm{nm}^{-2}$	$c\,/\,\mathrm{k_B T}\cdot\mathrm{nm}^{-1}$	$d\,/\,\mathrm{k_B T}$	$\Delta U\,/\,\mathrm{k_B T}$	$U''_{\mathrm{A,B}}\,/\,\mathrm{k_B T}\cdot\mathrm{nm}^{-2}$
6.13	24.15	-19.29	4.93	113.26	± 61.26

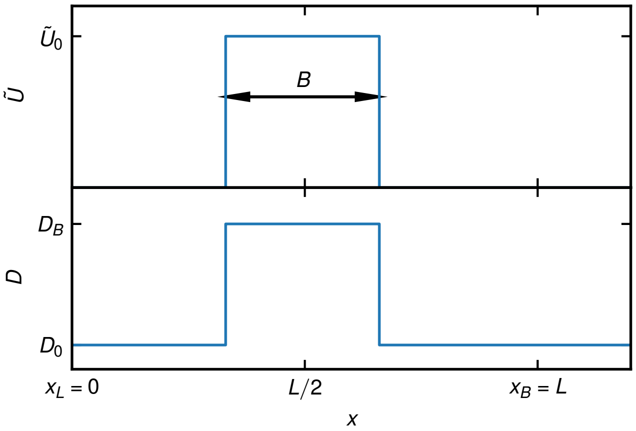

Supplementary Figure 8. Schematic representation of the box-like potential and diffusivity profiles with a width B.

In the main text, we model the potential barrier of the air-water interface as a single well using a cubic potential

$$U(z, F_{\mathrm{pull}}) = az^3 + bz^2 + (c + F_{\mathrm{pull}})z + d\,, \tag{37}$$

where a, b, c and d are fit parameters and F_{pull} is the applied force. The extrema of Supplementary Equation (37) are obtained by $\partial U(z)/\partial z = 0$ and given as

$$z_{\mathrm{A,B}} = \frac{-b \mp \sqrt{b^2 - 3a(c + F_{\mathrm{pull}})}}{3a}\,. \tag{38}$$

From the extrema, we obtain the barrier height as

$$\Delta U = \frac{4\left[b^2 - 3a(c + F_{\mathrm{pull}})\right]^{3/2}}{27a^2}\,, \tag{39}$$

and the curvature as

$$U''_{\mathrm{A,B}} = \pm 2\sqrt{b^2 - 3a(c + F_{\mathrm{pull}})}\,. \tag{40}$$

Supplementary Table I shows the optimal fit parameters of the polynomial fit shown in Fig. 3 in the main text. Additionally we also provide the values for ΔU and $U''_{\mathrm{A,B}}$.

Supplementary Section 6. Reaction rates are dominated by the transition-state diffusivity

In the following, we demonstrate that the reaction rate is primarily determined by the transition-state diffusivity. For this, we consider a box-like potential profile with a barrier height $\tilde{U}_0$ and width B and a diffusivity profile that exhibits at the barrier a diffusivity D_B and otherwise a diffusivity D_0. See Supplementary Figure 8 for a schematic representation of the potential and diffusivity profiles. The mean first-passage time τ_{MFP} for starting at a position $x = 0$ and reaching the position $x = L$ for the first time is equivalently to Supplementary Equation (23) given by

$$\tau_{\mathrm{MFP}} = \int_0^L \mathrm{d}x \frac{\mathrm{e}^{\tilde{U}(x)}}{D(x)} \int_0^x \mathrm{d}\tilde{x}\mathrm{e}^{-\tilde{U}(\tilde{x})}\,. \tag{41}$$

We now split the integral into three parts, to the left of the barrier $0 < x < (L-B)/2$, at the barrier $(L-B)/2 < x < (L+B)/2$ and to the right of the barrier $(L+B)/2 < x < L$. The resulting mean first-passage time reads

$$\tau_{\mathrm{MFP}} = \int\limits_{0}^{\frac{L-B}{2}} \mathrm{d}x \frac{1}{D_0} \int_0^x \mathrm{d}\tilde{x} + \int\limits_{\frac{L-B}{2}}^{\frac{L+B}{2}} \mathrm{d}x \frac{\epsilon}{D_B} \int_0^x \mathrm{d}\tilde{x} \mathrm{e}^{-\tilde{U}(x)} + \int\limits_{\frac{L+B}{2}}^{L} \mathrm{d}x \frac{1}{D_0} \int_0^x \mathrm{d}\tilde{x} \mathrm{e}^{-\tilde{U}(x)}\,, \tag{42}$$

where we introduced the abbreviation $\epsilon := \exp(\tilde{U}_0)$. Evaluating the three integrals lead to

$$\tau_{\mathrm{MFP}} = \frac{L^2}{2D_0} + \frac{LB}{2}\left(\frac{1}{DB} - \frac{1}{D_0}\right) + \frac{B(L-B)}{2}\left(1 - \frac{1}{\epsilon}\right)\left(\frac{\epsilon}{DB} - \frac{1}{D_0}\right). \tag{43}$$

In the limit of a high barrier $\epsilon \to \infty$ the mean first-passage time simplifies to

$$\lim_{\epsilon\to\infty} \tau_{\mathrm{MFP}} \approx \frac{B(L-B)\epsilon}{2D_B}\,, \tag{44}$$

which shows that for high barrier the mean first-passage time is dominated by the diffusion constant at the barrier, which corresponds to the transition-state diffusivity.

SUPPLEMENTARY REFERENCES

[1] S. Miyamoto and P. A. Kollman, Settle: An analytical version of the SHAKE and RATTLE algorithm for rigid water models, Journal of Computational Chemistry 10.1002/jcc.540130805 (1992).

[2] I.-C. Yeh and M. L. Berkowitz, Ewald summation for systems with slab geometry, The Journal of Chemical Physics http://dx.doi.org/10.1063/1.479595 (1999).

[3] D. E. Smith and L. X. Dang, Computer simulations of NaCl association in polarizable water, The Journal of Chemical Physics 10.1063/1.466363 (1994).

[4] G. Bussi, D. Donadio, and M. Parrinello, Canonical sampling through velocity rescaling, The Journal of Chemical Physics 10.1063/1.2408420 (2007).

[5] G. C. Boulougouris, I. G. Economou, and D. N. Theodorou, Engineering a Molecular Model for Water Phase Equilibrium over a Wide Temperature Range, J. Phys. Chem. B 10.1021/jp972582l (1998).

[6] D. R. Lide, *CRC Handbook of Chemistry and Physics, 85th Edition* - (CRC Press, Boca Raton, Fla, 2004).

[7] P. V. Klimovich, M. R. Shirts, and D. L. Mobley, Guidelines for the analysis of free energy calculations, J Comput Aided Mol Des 10.1007/s10822-015-9840-9 (2015).

[8] T. C. Beutler, A. E. Mark, R. C. van Schaik, P. R. Gerber, and W. F. van Gunsteren, Avoiding singularities and numerical instabilities in free energy calculations based on molecular simulations, Chemical Physics Letters 10.1016/0009-2614(94)00397-1 (1994).

[9] J. D. Chodera, W. C. Swope, J. W. Pitera, C. Seok, and K. A. Dill, Use of the Weighted Histogram Analysis Method for the Analysis of Simulated and Parallel Tempering Simulations, J. Chem. Theory Comput. 10.1021/ct0502864 (2007).

[10] M. N. Tamashiro and M. A. Constantino, Ions at the Water-Vapor Interface, J. Phys. Chem. B 10.1021/jp911898t (2010).

[11] P. Loche, C. Ayaz, A. Schlaich, D. J. Bonthuis, and R. R. Netz, Breakdown of Linear Dielectric Theory for the Interaction between Hydrated Ions and Graphene, J. Phys. Chem. Lett. 10.1021/acs.jpclett.8b02473 (2018).

[12] J. S. Hub, B. L. de Groot, and D. van der Spoel, G_wham—A Free Weighted Histogram Analysis Implementation Including Robust Error and Autocorrelation Estimates, J. Chem. Theory Comput. 10.1021/ct100494z (2010).

[13] H. Risken, *The Fokker-Planck Equation* (Springer Berlin Heidelberg, 1984).
