# Supplementary material for: Molecular dynamics simulations of the evaporation of hydrated ions from aqueous solution
Source: Commun Chem. 2022 Apr 19;5:55. doi: 10.1038/s42004-022-00669-5 (PMC9814746; doi:10.1038/s42004-022-00669-5)
Supplement: Supplementary file 3 — Description of Additional Supplementary Files [file 42004_2022_669_MOESM3_ESM.pdf]

Description of Additional Supplementary Files

File Name: Supplementary Movie 1

Description: Classical molecular dynamics simulation of a chloride ion pulled through an vapor-liquid water interface with a constant velocity of 300 m/s.

File Name: Supplementary Movie 2

Description: Simulation of the evaporation process of a chloride ion from the vapor-water interface. An externally applied constant force of 44 kBT/nm pulls the ion from the liquid water phase to the vapor phase.
